# Cosmosiin Induces Apoptosis in Colorectal Cancer by Inhibiting PD-L1 Expression and Inducing ROS

**DOI:** 10.3390/antiox12122131

**Published:** 2023-12-18

**Authors:** Jung Ho Han, Eun-Ji Lee, Wonyoung Park, Jang-Gi Choi, Ki-Tae Ha, Hwan-Suck Chung

**Affiliations:** 1 Korean Medicine (KM)-Application Center, Korea Institute of Oriental Medicine (KIOM), Daegu 41062, Republic of Korea; hanjh1013@kiom.re.kr (J.H.H.); jistr@kiom.re.kr (E.-J.L.); jang-gichoi@kiom.re.kr (J.-G.C.); 2 Department of Korean Medical Science, School of Korean Medicine, Pusan National University, Yangsan 50612, Republic of Korea; jinling0122@pusan.ac.kr (W.P.); hagis@pusan.ac.kr (K.-T.H.); 3 Korean Convergence Medical Science Major, University of Science and Technology (UST), KIOM Campus, Daegu 41062, Republic of Korea

**Keywords:** cosmosiin, PD-L1, cancer, reactive oxygen species, oxidative stress, apoptosis

## Abstract

Immunotherapies, particularly those concerning immune checkpoint inhibitors, have transformed cancer treatment in recent years. Programmed death-ligand 1 (PD-L1) is a key target for immunotherapy that is overexpressed in the cells of colorectal cancer, a widespread malignant cancer that poses a significant healthcare challenge. This study investigated the effects of cosmosiin treatment on colorectal cancer cell lines. Cosmosiin is a naturally occurring flavone glycoside compound that has potential health benefits, including antioxidant and immunomodulatory effects. This study showed that cosmosiin effectively suppresses the expression of PD-L1 and triggers apoptosis, which is facilitated through pathways that are related to reactive oxygen species. These outcomes suggest that cosmosiin could be a promising candidate for an immune checkpoint inhibitor in the treatment of colorectal cancer.

## 1. Introduction

Colorectal cancer (CRC) is among the most common forms of malignancy worldwide, imposing a significant global health burden [1]. Although there have been advances in its diagnosis and treatment, CRC remains a formidable challenge, requiring the further exploration of novel therapeutic strategies [2]. Immunotherapy, in particular approaches involving immune checkpoint inhibitors (ICIs), has transformed cancer treatment in recent years, showing remarkable success in treating a range of tumor types [3]. One key target in immunotherapy is programmed death-ligand 1 (PD-L1), a cell-surface protein that plays a pivotal role in evading immune surveillance and promoting tumor immune escape [4]. PD-L1 expression in tumor cells and immune cells in the tumor microenvironment has been found to be associated with immune evasion and disease progression in several malignancies [5]. As understanding of the complex interplay between cancer cells and the immune system grows, the investigation of the link between colon cancer and PD-L1 expression has emerged as a compelling area of research, with potential clinical implications [6].

Cosmosiin (COS) has synonyms, including apigetrin and apigenin-7-glucoside, a naturally occurring flavone glycoside compound [7,8]. COS has been used in traditional medicine systems for its health benefits, and its chemical structure has revealed that it possesses pharmacological properties that have therapeutic implications [8,9,10,11]. Preliminary research has suggested that this compound exhibits a wide array of bioactivities, including antioxidant and immunomodulatory activities. Notably, this compound was among the primary constituents of *Salvia plebeia*, a medicinal herb employed in prior investigations [12]. The growing demand for safe and effective therapeutic agents has led to an increased focus on alternative treatments, including the study of natural compounds such as COS [13,14]. COS has significant promise as a potential pharmaceutical agent due to its intriguing chemical structure and its demonstrated pharmacological activities [15]. Understanding the mechanisms of its action and evaluating its safety profile are crucial steps for harnessing its therapeutic potential for various diseases. In this context, COS has emerged as a particularly intriguing natural compound with versatile pharmacological properties, which make it a compelling candidate for the modulation of PD-L1 expression in cancer cells. The overexpression of PD-L1 in cancer cells is implicated in immune evasion and tumor progression, making this an attractive target for a therapeutic intervention [16]. Within the realm of cancer immunotherapy, a burgeoning interest has arisen in exploring natural compounds as potential PD-L1 modulators [17]. These compounds, which are derived from a range of plant sources, possess an array of bioactive properties and are being extensively studied for their potential in cancer therapy [18]. This research investigated the impact of controlling PD-L1 expression in cancer, with a focus on the use of natural compounds as promising drug candidates for unlocking novel avenues in cancer treatment and improving patient outcomes.

Several articles have highlighted the significance of PD-L1 expression in cancer progression and have demonstrated its correlation with the efficacy of ICIs [19,20]. Interestingly, a growing body of evidence supports the notion that a single compound, acting as an ICI, can both induce reactive oxygen species (ROS) and trigger apoptosis in cancer cells concurrently [21]. These findings have sparked considerable interest in exploring the therapeutic implications of ROS-mediated apoptosis as a mechanism for modulating PD-L1 expression and enhancing the effectiveness of cancer immunotherapy [22,23]. This thesis builds upon these intriguing observations, investigating the interplay between ROS, apoptosis, and PD-L1 expression, with a specific focus on the use of a unique natural compound as a potential ICI in cancer treatment. However, there have not been any reports on the use of COS to regulate PD-L1 expression in CRC. In this study, we investigated the effects of COS treatment on PD-L1 expression and related pathways, such as ROS and apoptosis, to determine its potential as an ICI and a promising therapeutic agent for CRC.

## 2. Materials and Methods

### 2.1. Materials

A cell counting kit-8 (CCK-8) was purchased from Dojindo Molecular Technologies (#CK04, Rockville, MD, USA). COS was purchased from ChemFaces (#CFN98981, purity ≥ 98%, Wuhan, China). Human interferon-gamma (IFN-γ) Recombinant Protein was purchased from R&D Systems (#285-IF-100, purity ≥ 97%, Minneapolis, MN, USA). Crystal violet was purchased from Sigma-Aldrich (#V5265, St. Louis, MO, USA), and 2’,7’-dichlorodihydrofluorescein diacetate (DCFH-DA) was purchased from Thermo Fisher Scientific (#D399, Waltham, MA, USA). Antibodies against PD-L1, p-AKT, AKT, p-ERK, ERK, PARP, and cleaved-PARP were purchased from Cell Signaling Technology (#13684S, #4060S, #4691S, #4370S, #4695S, #9542S, #9541S, Danvers, MA, USA). Antibodies targeting B-cell lymphoma-2 (Bcl-2) and Bcl-2-associated X (BAX) were acquired from Novus Biologicals (#NBP2-34443, #NBP1-88682, Littleton, CO, USA). Antibodies against glycoceramide 3-phosphate dehydrogenase (GAPDH) were purchased from Santa Cruz Biotechnology (#sc-47724, Santa Cruz, CA, USA).

### 2.2. Cell Culture

DLD-1, Hct116, RKO, and Jurkat T cells were purchased from the American Type Culture Collection (Manassas, VA, USA). DLD-1, Hct116, and RKO cells are human colorectal cancer cell lines, and Table 1 displays the gene types of representative oncogenes [24,25,26]. DLD-1 and Jurkat T cells were cultured in the Roswell Park Memorial Institute 1640 Medium (RPMI 1640), and Hct116 and RKO cells were cultured in Dulbecco’s modified Eagle’s medium containing 10% heat-inactivated fetal bovine serum and 1% penicillin/streptomycin. All of the cells were cultured in a humidified CO_2_ incubator at 37 °C and 5% CO_2_. Cell-culture-related solutions were purchased from Hyclone Laboratories, Inc. (GE Healthcare Life Sciences, Chicago, IL, USA).

### 2.3. Cell Viability Assay

The cell viability assay was conducted using a CCK-8 assay. The DLD-1, RKO, and Hct116 cells were seeded in a 96-well plate at a density of 5 × 10^3^ cells/well and were cultured with the specified dose of COS for the designated duration. After the treatment period, the CCK-8 reagent was added, following the manufacturer’s protocol. The cells were then incubated for 1–2 h at 37 °C and at 5% CO_2_ in a cell culture incubator. The absorbance was measured at 450 nm with a Spectramax M2 Microplate Reader (Molecular Devices, Sunnyvale, CA, USA).

### 2.4. Western Blot Analysis

The cells were rinsed using phosphate-buffered saline (PBS), and the total cellular proteins were extracted using a RIPA buffer along with a 1% NP-40 lysis buffer, including protease inhibitor cocktail tablets (Roche, Basel, Switzerland). The protein levels in the samples were measured using the Bio-Rad protein assay. The uniform protein quantities were separated using 8–15% SDS-PAGE in all samples, followed by the electrophoretic transfer of proteins onto nitrocellulose membranes (GE Healthcare, Munich, Germany). Following the blocking of the membranes at room temperature (20–25 °C) for 1 h with 1–5% nonfat dry milk or 1–5% bovine serum albumin, they were subjected to overnight incubation with primary antibodies at 4 °C. Following this, the membranes underwent washing three times with Tris-buffered saline for 10 min each. The Bio-Rad Chemidoc imaging system (Bio-Rad, Hercules, CA, USA) was employed to measure the specific protein bands. The protein expression levels were normalized against GAPDH.

### 2.5. Flow Cytometry

PD-L1 expression and apoptotic cell analysis were conducted using flow cytometry with APC anti-human CD274 antibody (B7-H1, PD-L1; #393610, BioLegend, San Diego, CA, USA) and a FITC Annexin V Apoptosis Detection Kit I (#556547; BD Biosciences, San Jose, CA, USA), respectively. The cells were stained with the antibody and an Annexin V-FITC Apoptosis Detection Kit following the manufacturer’s instructions. The fluorescence of stained cells was measured in the FITC and PE channels, gating the living and apoptosis cells. The quantification of PD-L1 expression and the ratio of apoptotic cells were assessed using a CytoFLEX flow cytometer (Beckman Coulter Inc., Pasadena, CA, USA).

### 2.6. Co-Culture Experiments

Jurkat T cells transduced with a PD-1-expressing lentivirus were utilized, and PD-1 expression has been confirmed via FACS analysis in a previous study [10]. Jurkat T cells were activated using human activator beads (Dynabeads™ Human T-Activator CD3/CD28, Thermo Fisher Scientific, Waltham, MA, USA). The cancer cells were seeded at a density of 1 × 10^5^ cells in 12-well plates and were allowed to attach overnight. Then, a pretreatment with COS at the indicated dose was administered for 4 h, followed by the addition of 20 ng/mL of IFN-γ. The activated Jurkat T cells were cocultured with cancer cells over a 2-day period, during which the coculture was incubated at 37 °C and 5% CO_2_ in a cell culture incubator. Following the 2-day coculture, the supernatants were collected for cytokine measurements. The plates were washed with PBS, and the cells were stained using a crystal violet solution. Following staining, the crystal violet solution was removed, the plates were dried, and the extent of staining was quantified.

### 2.7. Cytokine Measurement

The medium was obtained from the coculture experiments. Then, the cells were removed by centrifuge, and cell-free media were obtained. The level of interleukin-2 (IL-2, #555,148, BD Biosciences, San Jose, CA, USA) in the cell-free media was measured using an ELISA kit. Each ELISA kit was measured according to the manufacturer’s instructions.

### 2.8. Determination of Intracellular Reactive Oxygen Species

The intracellular ROS levels were assessed using flow cytometry assays. Cells were seeded in six-well plates at a density of 1–5 × 10^5^ cells per well and were subjected to treatment with the indicated concentrations of COS and N-acetylcysteine (NAC). Following a 2-day incubation period, the cells were washed three times with prewarmed 1 xPBS. A working solution of 0.1 µM DCFH-DA in PBS was prepared, shielded from light, and then incubated at 37 °C for 30 min. Following incubation, cells were washed three times with prewarmed DPBS and subsequently resuspended. The levels of ROS were quantified using a CytoFLEX flow cytometer. Intracellular ROS fluorescence images were obtained with a fluorescence microscope (Nikon ECLIPSE Ti-U, Nikon Co., Tokyo, Japan), and intensity was interpreted as the intracellular ROS level.

### 2.9. Statistical Analysis

The data for cell viability and PD-L1 expression were obtained by flow cytometry assays and coculture experiments, and intracellular ROS was expressed as ratios relative to the corresponding control values. The results were presented as the means ± standard errors of the mean (SEM) from three experimental replicates. Variations between the mean values within each group were analyzed using a Student’s *t*-test. The differences among multiple groups were assessed using a one-way analysis of variance, followed by a Tukey’s post hoc examination. All statistical analyses were performed using GraphPad Prism version 8.0 (GraphPad Software, San Diego, CA, USA) to ensure accurate data-handling practices.

## 3. Results

### 3.1. Cell Viability on Colon Cancer Cells by COS Treatments

**Figure 1:**
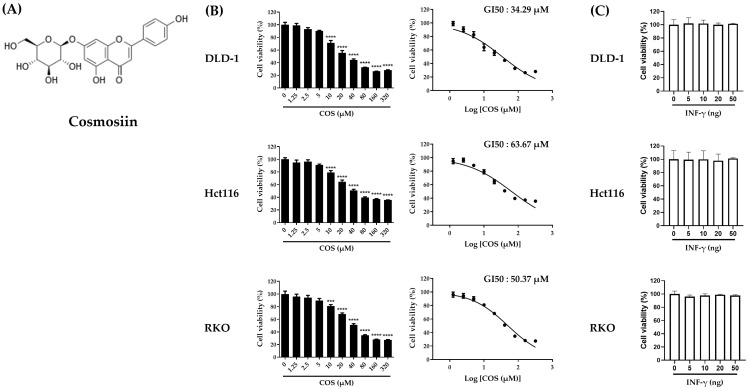
Cell viability, measured by COS and IFN-γ treatment in colon cancer cell lines. (**A**) Structural formula of COS. (**B**,**C**) Cell viability was assessed for DLD-1, Hct116, and RKO cells following exposure to COS and IFN-γ at the specified doses over a period of 2 days. The viabilities of three cells were analyzed by CCK. The results are shown as the means and SEMs. *** *p* < 0.001 and **** *p* < 0.0001 relative to the respective controls. The experiments were conducted independently three times.

Figure 1A illustrates the structural formula of COS, a compound pivotal to our study. Cell viability assays were performed to determine the toxicity and effectiveness of COS on colon cancer cells. The colorectal cell lines DLD-1, RKO, and Hct116 were treated with COS at the indicated doses for 2 days. After treatment, COS significantly decreased cell viability in all three cell lines. The 50% growth inhibition (GI50) values were 34.29 µM, 63.67 µM, and 50.37 µM for DLD-1, RKO, and Hct116 cells, respectively (Figure 1B). By contrast, no significant difference in cell viability was observed with the IFN-γ treatment for 2 days (Figure 1C).

### 3.2. COS Inhibits the PD-L1 Expression of Colon Cancer Cells

**Figure 2:**
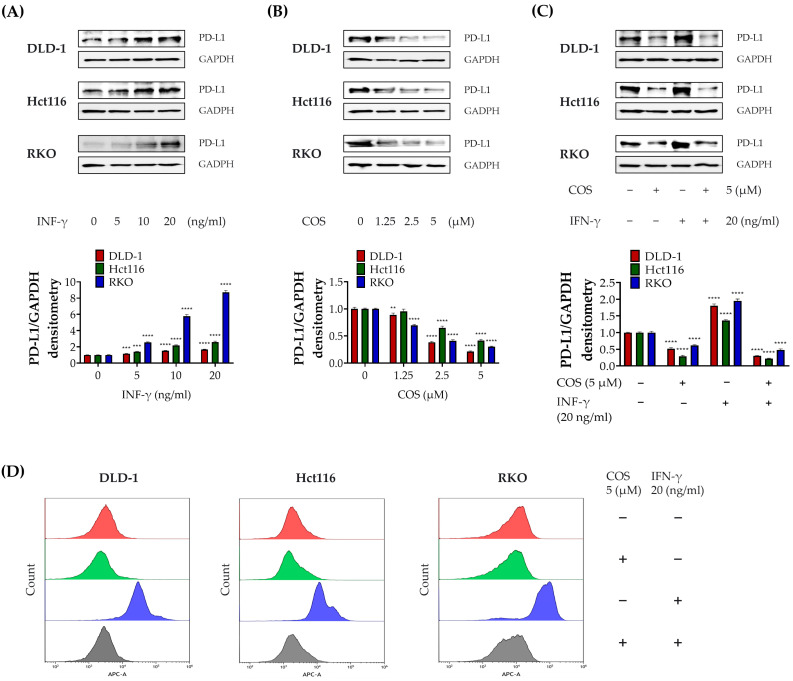
COS suppresses the PD-L1 expression in a colon cancer cell line. (**A**–**D**) The PD-L1 expression of DLD-1, Hct116, and RKO cells was measured following treatment with a COS and IFN-γ indicated dose for 2 days. Experiments (**A**–**C**) and (**D**) were performed with a Western blot assay and FACS, respectively. The results are shown as the means and SEMs. ** *p* < 0.01, *** *p* < 0.001, and **** *p* < 0.0001 relative to the respective controls. The experiments were conducted independently three times.

We have previously shown that *Salvia plebeia* R. Br. extract and its major compound COS blockade PD-1/PD-L1 interaction [10]. We hypothesized that COS would have the function of inhibiting PD-L1 expression in colon cancer cells. From the results in Figure 1, we determined the treatment conditions of COS (5 µM) and IFN-γ (20 ng/mL) and confirmed changes in PD-L1 expression using Western blot assay. As reported elsewhere, IFN-γ expression increases in a dose-dependent manner, while COS expression was suppressed (Figure 2A,B). When COS and IFN-γ were treated in combination, PD-L1 expression was reduced (Figure 2C). The same results were seen when measured with FACS (Figure 2D). These results suggest that COS suppresses PD-L1 expression.

**Figure 3:**
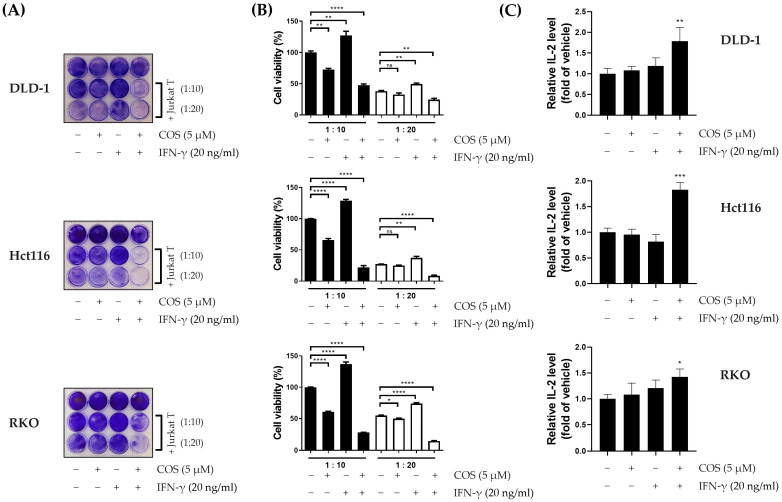
COS augmentation of T-cell-mediated cell death and immune mediator release in a colon cancer cell line. (**A**,**B**) Coculture experiments were conducted in seeded colon cancer cell lines with added COS (5 μM), IFN-γ (20 ng), and Jurkat T cells (1:10, 1:20) for 24 h. After incubation, the cancer cells were stained with crystal violet, and the intensity of live cells was measured and bar graphed. (**C**) The level of IL-2 in the coculture medium was estimated using an ELISA kit. The viabilities and cytokines were examined individually, using CCK and ELISA. The results are shown as the means and SEMs. * *p* < 0.05, ** *p* < 0.01, *** *p* < 0.001, **** *p* < 0.0001, and ns (not significant) compared to their respective controls. The experiments were conducted three times independently.

Considering the critical role of PD-L1 expression in cancer cell immune escape, our study investigated the impact of COS on T-cell-mediated cell death in colon cancer cells, based on its observed effect (Figure 2) on PD-L1 expression regulation. To achieve this, we conducted a series of viability assays involving DLD-1, RKO, and Hct116 colon cancer cells, using Jurkat T cells that expressed PD-1 [10]. These colon cancer cells were subjected to various treatments, including individual exposures to COS (5 µM) and IFN-γ (20 ng/mL), as well as cotreatments involving Jurkat T cells at ratios of 1:10 and 1:20. Our experimental results indicated several important trends. IFN-γ treatment was associated with increased cell viability, while COS treatment led to reduced viability both when administered individually and when combined with other treatments (Figure 3A). Notably, the viability of colon cancer cells exhibited a decreasing trend as the proportion of cocultured Jurkat T cells increased. Cell viability data were presented in the form of a bar graph (Figure 3B). To obtain further insight, we also conducted assessments of IL-2 levels in the culture media of coculture experiments. Encouragingly, we also observed an elevated concentration of this immune-related factor during cotreatment with COS and IFN-γ (Figure 3C). These collective findings provide compelling evidence that COS plays a significant role in enhancing T-cell-mediated cell death by suppressing PD-L1 expression, diminishing the immunosuppressive capacity of the PD-1/PD-L1 axis.

### 3.3. COS Induces Apoptosis by Downregulating AKT and ERK Signaling in Colon Cancer Cells

**Figure 4:**
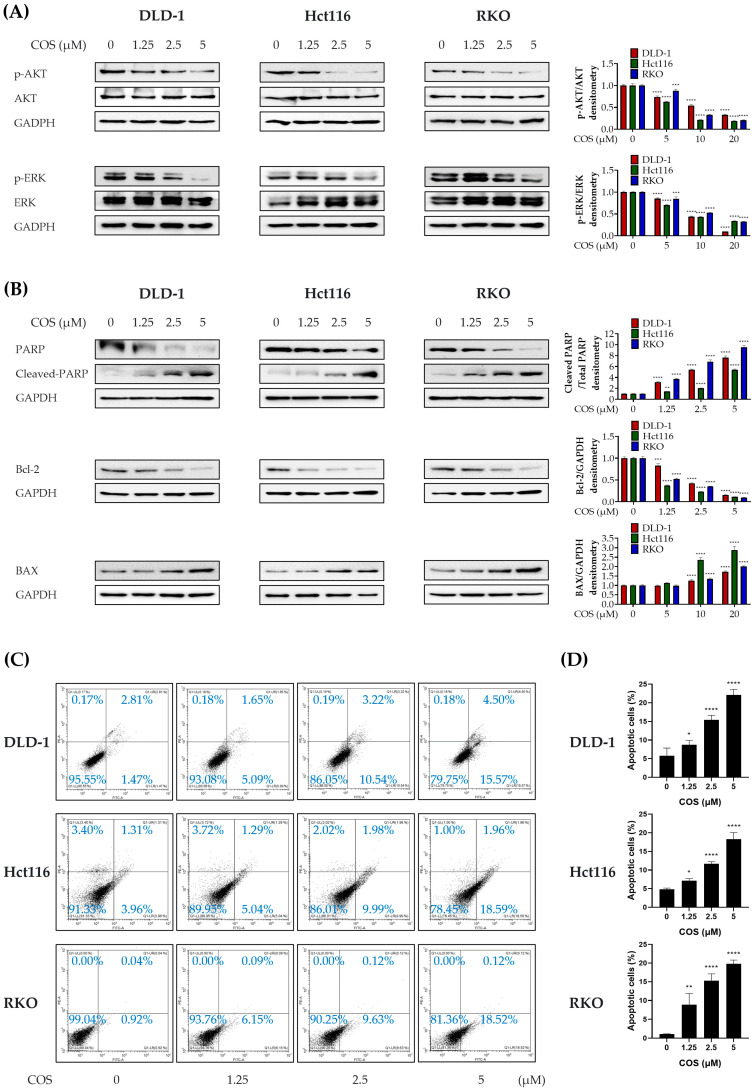
Measurement of apoptosis-related protein and the ratio of apoptotic cells in a colon cancer cell line by COS. (**A**,**B**) Protein expression levels for p-AKT, AKT, p-ERK, ERK, PARP, cleaved-PARP, Bcl-2, BAX, and GAPDH were assessed using Western blot analysis. (**C**) The quantification of apoptotic cells was conducted through FACS analysis, employing PI-Annexin V staining. (**D**) The bar chart represents the cells of the ratio of apoptosis. DLD-1, Hct116, and RKO cells were treated with COS (0, 1.25, 2.5, or 5 μM) for a duration of 2 days. The results are shown as the means and SEMs. * *p* < 0.05, ** *p* < 0.01, *** *p* < 0.001, and **** *p* < 0.0001 compared to their respective controls. The experiments were conducted three times independently.

The data shown in Figure 2 confirm the reduction in PD-L1 expression following COS treatment. Subsequently, we investigated the underlying pathway that is responsible for this effect. Following the established knowledge, which suggests that the inhibition of the PI3K–AKT–mTOR pathway leads to decreased PD-L1 expression and instigates PD-L1 upregulation via the ERK signaling pathway, we evaluated the expression levels of AKT and ERK [27,28]. Our data indicate that COS (0, 1.25, 2.5, 5 µM) treatment dose-dependently decreases p-AKT and p-ERK in colon cancer cells (Figure 4A). In accordance with the observation that ERK and AKT signaling pathways are downregulated in colon cancer cells to facilitate apoptosis, our study also assessed the apoptosis signaling pathway [29]. The expression patterns of apoptosis-related proteins, including PARP, Bcl-2, and BAX, demonstrate that COS (0, 1.25, 2.5, 5 µM) treatment leads to the induction of apoptosis (Figure 4B). Then, the occurrence of apoptosis was measured by FACS. It was confirmed through Annexin V–FITC/PI staining that the apoptosis extent increased in a concentration-dependent manner with COS (0, 1.25, 2.5, 5 µM) (Figure 4C,D). Taken together, these results show that COS induces apoptosis through the regulation of AKT and ERK signaling.

### 3.4. COS Treatment Increases ROS Levels in DLD-1, Hct116, and RKO Cells

**Figure 5:**
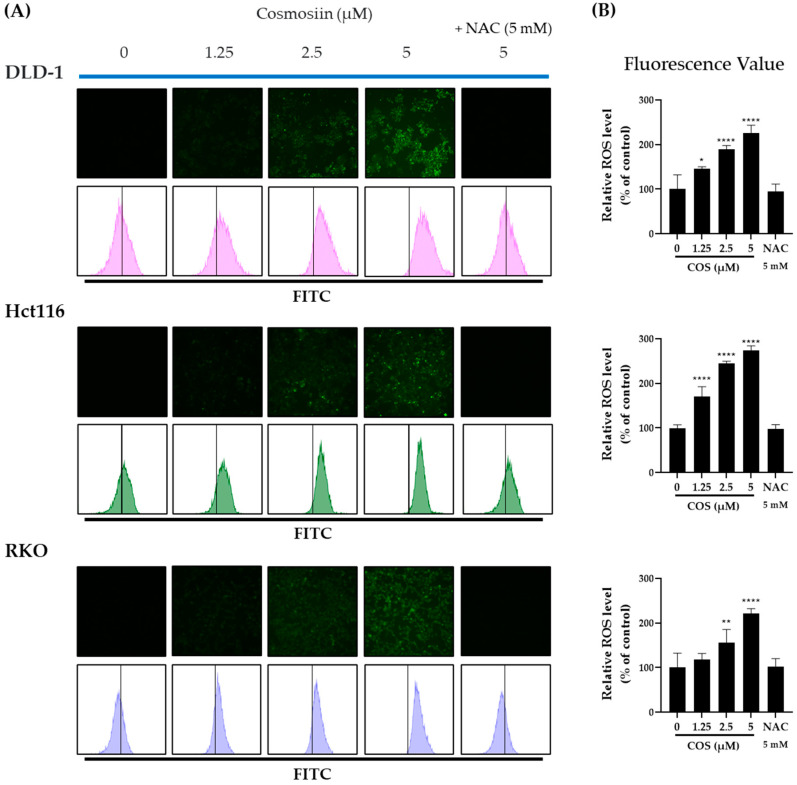
Increased production of ROS in DLD-1, Hct116, and RKO cells by COS treatment. (**A,B**) DLD-1, Hct116, and RKO cells were treated with the indicated COS and NAC for 2 days, and ROS was measured using FACS. The bar graphs on the right present the fluorescence values and FITC mean values, respectively. * *p* < 0.05, ** *p* < 0.01, and **** *p* < 0.0001 compared to their respective controls. The experiments were conducted three times independently.

Several studies have investigated the relationship between reactive oxygen species (ROS) and PD-L1 expression [21]. Among the drugs producing ROS in cancer cells, some have been shown to up- or downregulate PD-L1 [21]. The role of ROS in triggering apoptosis is widely acknowledged in various publications [30]. Because we observed apoptosis resulting from COS treatment in our prior experiment, we formulated the hypothesis that COS treatment induces apoptosis by generating ROS. Consequently, we quantified ROS levels. Measurements using fluorescence and flow cytometry showed a concentration-dependent increase in ROS levels in DLD-1, Hct116, and RKO cells following COS (0, 1.25, 2.5, 5 µM) treatment, which was reversed by NAC treatment (Figure 5A). Fluorescence values were represented in a bar graph (Figure 5B).

## 4. Discussion

In recent years, ICIs have emerged as a part of groundbreaking therapies in oncology, revolutionizing the treatment landscape for a range of cancers [31]. The regulation of PD-L1 expression has immense significance for ICI therapy [32]. For maximizing the efficacy of ICIs and optimizing patient outcomes, understanding the intricate mechanisms that govern PD-L1 expression is pivotal [33]. PD-L1 expression levels in tumor tissues have been linked with patient responsiveness to ICIs, making it a predictive biomarker for treatment success [16]. Tumors with higher PD-L1 expression tend to exhibit a greater immunosuppressive microenvironment, rendering them more susceptible to an immune checkpoint blockade [34]. At present, extensive research efforts are being directed toward unraveling the complex regulatory pathways that control PD-L1 expression [35]. Multiple factors influence PD-L1 expression, including oncogenic signaling pathways, inflammatory cytokines, genetic alterations, and epigenetic modifications [36]. For instance, oncogenic signaling pathways, including the MAPK and PI3K pathways, can upregulate PD-L1 expression in response to cellular stress and inflammation [37]. In addition, tumor-infiltrating immune cells, such as interferon-gamma-producing T cells, can induce PD-L1 expression in cancer cells through a positive feedback loop [38]. Efforts are also underway to develop strategies of modulating PD-L1 expression and enhancing the effectiveness of ICIs [39]. Combination therapies targeting both PD-L1 expression and other immune checkpoints or signaling pathways are being explored to overcome resistance mechanisms and broaden patient populations that can benefit from ICIs [40]. Additionally, understanding the role of PD-L1 expression dynamics in disease progression and treatment response is crucial for tailoring therapeutic approaches on an individualized basis.

This study investigated the use of COS as a regulator of PD-L1 expression in CRC, a novel and promising avenue within cancer immunotherapy. This investigation aligns with an increasing interest in natural compounds as potential modulators of immune responses, harnessing their diverse bioactive properties for therapeutic benefit [41,42]. The investigation into the effects of COS treatment on CRC cell lines provided two crucial findings, namely, the inhibition of PD-L1 expression and the induction of apoptosis through ROS-related pathways. The observed inhibition of PD-L1 expression for COS treatment aligns with the growing interest in natural compounds as potential PD-L1 modulators [43,44]. COS’s ability to downregulate PD-L1 expression has implications for the disruption of the immune evasion mechanisms employed by cancer cells, potentially enhancing the effectiveness of approaches using immunotherapy. Furthermore, the induction of apoptosis through ROS-related pathways forms a significant mechanistic insight [45]. The connection between apoptosis and immune checkpoint regulation opens an intriguing perspective [46]. ROS-mediated apoptosis not only affects cancer cell survival but also has potential implications for modulating the immunogenicity of the tumor microenvironment [47,48]. This study’s contribution to elucidating the interplay among ROS, apoptosis, and PD-L1 expression contains valuable insights into the potential synergistic mechanisms underlying cancer immunotherapy. Further investigation is warranted to validate the observed effects across a broader range of CRC models and to more comprehensively delineate the molecular pathways that underlie COS’s actions. Additionally, the assessment of COS’s safety profile and its potential interactions with other therapeutic agents will be critical steps for translating these findings into clinical applications.

Previous studies on *Salvia plebeia* in our laboratory have shown that COS possesses immunotherapeutic properties, including its demonstration of the most potent PD-1/PD-L1 blockade, functioning as an ICI [10]. This study further substantiates the finding that COS directly inhibits PD-L1 expression in colon cancer cell lines, producing an anticancer effect. Because in vivo experiments were carried out in preceding work, additional in vivo experiments were not performed here. However, future research, especially involving the exploration of novel treatment strategies, including combination treatments, necessitates the implementation of new in vivo tests.

Previous studies have established that the AKT and ERK signaling pathways contribute to the upregulation of PD-L1 expression in non-small cell lung cancer, multiple myeloma, and triple-negative breast cancer [27,49,50,51]. On the other hand, reports have indicated that PD-L1 can activate MEK/ERK and PI3K/AKT signaling pathways within CRC cells [52]. Our findings indicated a reduction in the levels of phosphorylated AKT and ERK (p-AKT, p-ERK) in CRC cells following treatment with COS, as depicted in Figure 4A. Because we used colon cancer cells in this paper, it may be that COS treatment downregulates AKT and ERK signaling through the regulation of PD-L1 expression, as is described in previous publications on CRC. IFN-γ regulates the transcription of PD-L1, leading to an increase in its expression [53,54]. However, ROS suppresses PD-L1 expression via the inhibition of fibroblast growth factor receptor 1 expression, the inhibition of the phosphorylation of eukaryotic translation initiation factor 4E-binding protein 1, and the activation of eukaryotic initiation factor 2α [22,55]. Consequently, Figure 2C demonstrates that although there is an increase in PD-L1 expression induced by IFN-γ, PD-L1 expression decreases due to elevated ROS resulting from COS treatment. However, the mechanism by which PD-L1 functions within CRCs remains unclear. ERK and AKT signaling pathways, as well as ROS and IFN-γ, involved in PD-L1 expression, require further investigation.

Several studies have indicated that drug treatments can result in increased ROS levels, leading to a decrease in PD-L1 expression. For instance, Ethaselen, an organoselenium compound, inhibits TrxR1, a redox sensor and antioxidant enzyme, which results in elevated ROS levels within cells and subsequently reduces the expression of PD-L1 [56,57]. Butaselen, a TrxR1 inhibitor and ROS generator, suppresses PD-L1 expression on the tumor cell surface through the STAT3 pathway [58,59]. Metformin, a biguanide drug commonly employed in diabetes treatment, has anticancer effects both in vitro and in vivo [60]. It prompts cancer cell apoptosis through oxidative stress and ROS production [60,61]. Additionally, metformin reduces PD-L1 expression by modulating the Hippo signaling pathway [62]. Chaetocin, a potent inhibitor of TrxR1 and histone methyltransferase, triggers apoptosis in cancer cells by inducing excessive accumulations of ROS [63]. Notably, chaetocin treatment markedly diminishes PD-L1 protein levels in human pancreatic cancer cells [64]. On the other hand, numerous studies have demonstrated that drug usage can elevate ROS levels, resulting in augmented PD-L1 expression [65,66,67,68]. However, our findings align with those of other studies that have indicated a correlation between increased ROS levels and subsequent reductions in PD-L1 expression.

This study suggests that COS may have promising potential in conjunction with other cancer therapies, specifically ICIs. By inhibiting the expression of PD-L1, COS can sensitize cancer cells, making them more susceptible to the effects of ICIs. This synergistic approach can enhance treatment outcomes for cancer patients, although it is crucial to emphasize that further studies and clinical trials are imperative for substantiating this hypothesis [69]. These findings indicate the importance of exploring innovative combination therapies for augmenting the efficacy of cancer treatment and laying the groundwork for more effective strategies against cancer. However, it is important to acknowledge a primary limitation of our study, namely, the relatively small number of CRC cell lines utilized in testing COS. To validate our findings, it is imperative to conduct additional research that encompasses a broader spectrum of types of cancer. Furthermore, comprehensive investigations are required to elucidate the optimal COS treatment methods in the contexts of various cancers.

## 5. Conclusions

**Figure 6:**
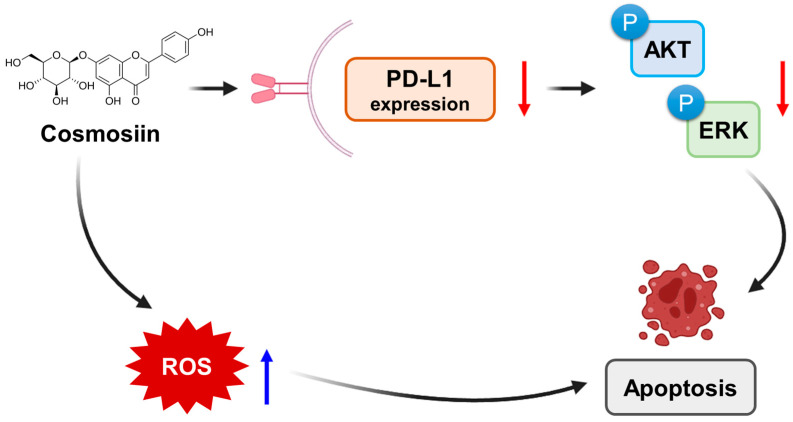
Schematic representation of the work on COS. COS inhibits the phosphorylation of AKT and ERK, and this inhibition leads to a decrease in the expression of PD-L1. Additionally, COS increases the production of ROS. These effects lead to the apoptosis of cancer cells.

This study demonstrates the potential of COS as an ICI in CRC treatment. Through regulating PD-L1 expression and initiating apoptosis via ROS-related pathways, COS is a naturally occurring compound with versatile effects that can bolster the effectiveness of immunotherapies. Further experimental and clinical validation is imperative for fully harnessing the therapeutic promise of COS to enhance patient outcomes in CRC and potentially other types of malignancies (Figure 6).

## Figures and Tables

**Table 1:** Gene types of human colorectal cancer cells.

Cell	KRAS	NRAS	BRAF
DLD-1	G13D	Wild type	Wild type
Hct116	G13D	Wild type	Wild type
RKO	Wild type	Wild type	V600E

## Data Availability

The information will be provided upon sensible inquiry.
